# Guesstimates are not good enough for determining what is happening in routine care

**DOI:** 10.1038/sj.bjc.6605998

**Published:** 2010-11-09

**Authors:** V Heong, S Ananda, J Tie, P Gibbs

**Affiliations:** 1 Department of Medical Oncology, Western Hospital; 2 BioGrid Australia; 3 Department of Medical Oncology, Royal Melbourne Hospital, Grattan Street, Parkville, Victoria 3050, Australia

**Sir,**

Shabaruddin *et al* (2010) recently reported on the results of a survey conducted by expert UK oncologists regarding their use by irinotecan for colorectal cancer, with the intent of using these data to explore the cost-effectiveness of routine UGT1A1 testing. Reading this article, we were struck with the wide variation in the experts’ answers, and wondered, as did the authors, as to how accurate the experts might be. To further explore this observation, we put the same questions to seven Melbourne (Australia) oncologists who work at four institutions. We then compared these responses with prospective data that they had collected on 604 patients with metastatic colorectal cancer, who had presented with stage IV disease, or developed recurrence, between 2005 and 2009.

We were initially struck by the similarity of the estimates made by the UK and Melbourne experts (Table 1) regarding treatment used in the first- to third-line setting. Examining the Melbourne data, it was evident that the Melbourne experts were overestimating the use of irinotecan, with even the lowest estimate of any expert being higher than real practice. In the third-line setting, regarding the use of mitomycin C, the discordance between estimates and real data was most striking (40% estimate versus 17.4% reality). This is consistent with clinicians reporting that they found it more difficult to estimate treatment use with increasing lines of therapy.

The Melbourne experts’ estimates for irinotecan duration in the second-line setting were quite accurate; however, in the first-line setting, in which all but one expert said either 5 or 6 months, the real data showed 3.2 months. The estimates of the incidence of febrile neutropenia ranged from 2 to 8%. Remarkably, the mean result was 5.4%, which is the exact figure found for the real data.

One possible explanation for the similarity between the UK and Melbourne estimates, in instances in which there are comparable data, is that both groups may be basing their answers on a common set of reference data, such as clinical trial results or other published data. Experts may then use this knowledge to derive their estimates, rather than have a true awareness of what is happening in their practice. To explore this possibility further, we compared the Melbourne experts’ estimates with available trial data. The duration of irinotecan use in first-line trials was 5.5–6.5 months (Douillard *et al*, 2000; Saltz *et al*, 2000; Tournigand *et al*, 2004) and that in second-line studies was 1.5–4.1 months (Cunningham *et al*, 1998; Tournigand *et al*, 2004; Sobrero *et al*, 2009; Gibbs *et al*, 2010), which was quite similar to the experts’ estimates (see Table 1). In two studies enrolling patients in a first-line setting, the percentage who then received second-line treatment was reported as 54 and 62% (Tournigand *et al*, 2004; Seymour *et al*, 2007), which is again similar to the experts’ estimates. The reported incidence of febrile neutropenia ranged from 2.1% (Douillard *et al*, 2000) to 7.1% (Sobrero *et al*, 2009), again in line with the experts’ estimates.

If the above explanation were true, we would also anticipate that experts’ estimates would be accurate when real data were similar to trial data, and inaccurate when, for whatever reason, there were significant differences between clinical studies and real life. This is most obvious for the duration of use of first-line irinotecan, in which further analysis of our hospital treatment data revealed that the dominant use of irinotecan in this setting was when patients had recently failed oxaliplatin-based adjuvant treatment, a poor prognosis group. This would explain the duration of first-line irinotecan treatment being similar to irinotecan use in the second-line setting, wherein patients typically receive treatment following failure of first-line oxaliplatin-based therapy. Although in retrospect this is an obvious explanation for the abbreviated first-line irinotecan-based treatment, it appears that only one of our experts may have factored this into their answers.

Finally, we also suggest that when there were no available trial data as a reference point, estimates were not only likely to be inaccurate but there was also a much wider variation in the answers provided by the experts. An example of such a query would be the use of third-line mitomycin C, or the reasons for discontinuing irinotecan. When these are examined, the data (Table 1) appear consistent with this observation. Estimates of third-line mitomycin C use ranged from 0 to 80%, and therapy completion as the reason for cessation of therapy ranged from 10 to 50% in both instances the means were quite different from the real data.

It is worth noting that when data are combined, as would be required for the analysis that Shabaruddin *et al* were undertaking, the inaccuracy of the experts’ opinions across the multiple data sets would be compounded. For example, regarding first-line irinotecan use, the amount of drug used per 100 patients in the Melbourne experts’ opinion would be 66 months (5 months duration in 13.2 patients per 100), a total that is 41.1 months or 3.49-fold greater than the real data (18.9 months=3.2 months for 5.9% of patients). Other estimates would be closer, including second-line irinotecan use, which parallels clinical trial data; others would be out by much more, such as the third-line use of mitomycin C.

Historically, for a speciality so driven by clinical trials that produce large amounts of data, we would agree with Shabaruddin *et al* that the scant amount of data regarding treatment and outcomes in routine care is surprising. Although collecting data is achievable, as we have demonstrated (see also http://www.biogrid.org.au), we acknowledge that it does require significant effort and resources. However, if understanding what is happening in routine care is important, and we believe it is for multiple reasons, then we would suggest that the necessary investments to support this must be made. On the basis of our small study, using the estimates of experts appears to be of limited value, and could be potentially quite misleading. These estimates may also become increasingly unreliable as treatment paradigms become more complex, use of biological therapy becomes more widespread and multiple lines of therapy become more common.

## Figures and Tables

**Table 1:** Survey results of seven medical oncologists, compared with real data from patients treated at four hospitals at which they work

**Query**	**Melbourne experts mean (range)**	**UK experts mean^a^**	**Clinical trial data**	**Melbourne data**
*First-line treatment*				
Oxaliplatin based	74.3% (55–90)	72.8%	N/A	70.1%
Irinotecan based	13.2% (7–20)	4.5%		5.9%
				
*Second-line treatment*				
Oxaliplatin based	15.1% (10–20)	17.7%		25.9%
Irinotecan based	69.8% (60–80)	63.7%	54–62%	54.2%
				
*Third-line treatment*				
Mitomycin C	40% (0–80)	50%	N/A	17.4%
				
*Duration*				
First-line irinotecan	5.0 months (3–6)	5 months^b^	5.5–6.5 months	3.2 months
Second-line irinotecan	3.3 months (2–4)		1.5–2.0 months	3.5 months
				
*Ceasing irinotecan*				
Therapy completion	26.5% (10–50)	25%^c^	N/A	40.3%
Disease progression	51.4% (40–70)	62%		39.6%
Treatment toxicity	15.26% (10–25)	32%		12.2%
Patient request	6.8% (0–15)	—		3.6%
Febrile neutropenia	5.4% (2–8)	8.4%	2.1–7.1%	5.4%

Abbreviation: N/A=not applicable.

a All oxaliplatin and irinotecan regimens combined.

b Not broken down by first- and second-line treatment.

c Numbers in survey do not add to 100%.
